# Vagal reactivation after a cardiac rehabilitation session associated with hydration in coronary artery disease patients: crossover clinical trial

**DOI:** 10.1038/s41598-021-89840-x

**Published:** 2021-05-18

**Authors:** Maria Júlia Lopez Laurino, Anne Kastelianne França da Silva, Lorena Altafin Santos, Felipe Ribeiro, Laís Manata Vanzella, Dayane Andrade Genoni Corazza, Luiz Carlos Marques Vanderlei

**Affiliations:** Physical Therapy Post-graduation Program, Faculty of Science and Technology, São Paulo State University (UNESP), Rua Roberto Simonsen, 305, Presidente Prudente, São Paulo 19060-900 Brazil

**Keywords:** Cardiology, Rehabilitation

## Abstract

This study aimed to investigate the hydration influence on the autonomic responses of coronary artery disease subjects in the immediate recovery period after a cardiovascular rehabilitation session, in view of the risks of a delayed autonomic recovery for this population. 28 males with coronary artery disease were submitted to: (I) Maximum effort test; (II) Control protocol (CP), composed by initial rest, warm-up, exercise and passive recovery; (III) Hydration protocol (HP) similar to CP, but with rehydration during exercise. The recovery was evaluated through the heart rate (HR) variability, HR recovery and by the rate of perceived exertion and recovery. The main results revealed that the vagal reactivation occurred at the first 30 s of recovery in HP and after the first minute in CP. A better behavior of the HR at the first minute of recovery was observed in HP. The rate of perceived exertion had a significant decrease in the first minute of recovery in HP, while in CP this occurred after the third minute. In conclusion, despite an anticipated vagal reactivation found at HP, these results should be analyzed with caution as there were no significant differences between protocols for all variables and the effect sizes were small.

## Introduction

Subjects with coronary artery disease (CAD) present an autonomic modulation impairment characterized by a decreased vagal activity and a sympathetic hyperactivity^1^, which is related to a risen risk of ischemic events, and a higher mortality rate^2^. Thus, the insertion of this population in cardiac rehabilitation programs (CRP) is essential, as physical exercise promotes positive modifications in the autonomic modulation^3^.

Despite the well-known physical exercise benefits, and although the rate of occurrence of major cardiovascular events during cardiac rehabilitation in stable patients is very low^4,5^, the post-exercise period represents a vulnerable state of the cardiovascular system, in which arrhythmias, syncope, and sudden death may occur^6,7^. During the early recovery phase, the autonomic nervous system (ANS) plays a central role in the cardiovascular deceleration, by rapidly decreasing the heart rate (HR) through vagal reactivation within the first minutes of recovery^8^. However, due to the sympathetic hyperactivity related to CAD, the vagal reentry is delayed in this population^9^. Thus, the HR, the cardiac workload and the myocardium oxygen consumption remain higher for a longer period, which favors the appearance of sudden events during the recovery^10^.

Given the risks related to a delayed vagal reactivation and HR reduction, the study of strategies capable of promoting a more efficient recovery on this population immediately after exercise is essential, as a faster recovery is related to a lower risk of sudden events^2^ and may contribute to the safety of CRP.

Considering that the fluid loss due to transpiration^11^ can negatively influence the ANS^12^ and directly affects the baroreflex mechanisms, contributing to the slower HR recovery after the exercise^13^, the fluid intake has been suggested as a strategy to accelerate the immediate^14–17^ and long-term^18–20^ autonomic and HR recovery after exercise. However, this topic has only been studied in healthy populations.

Thus, we highlight the importance of studying the influence of hydration in CAD subjects. Since this technic can be easily implemented in CRP as a strategy to decrease post-exercise risks, opening an important field of research in clinical practice, that may influence the current techniques used in these programs.

Considering these aspects, the study aimed to investigate the influence of hydration in the autonomic modulation of CAD patients, through HR recovery and HR variability, at the immediate recovery period of a cardiac rehabilitation session. Also, as secondary outcomes, its influence in the rate of perceived exertion, discomfort and recovery.

## Method

### Trial design and setting

This crossover clinical trial followed the Consolidated Standards of Reporting Clinical Trials (CONSORT) extension to randomized crossover trials^21^, and was registered at clinicaltrial.gov (NCT 03198806 – 26/06/2017).

The experimental procedure was divided into three phases with a minimal interval of 48 h between them and was performed at the Physical Therapy Study and Care Center—Presidente Prudente, São Paulo—Brazil. The phases were: Phase I. Cardiopulmonary Exercise Test; Phase II. Control Protocol (CP); and Phase III. Hydration Protocol (HP).

The volunteers were initially oriented to maintain their habitual physical activity habits while participating in the study, but to avoid vigorous physical activity and consumption of stimulant substances for 24 h before each phase, and to consume a light meal two hours before each phase^22^.

All procedures were approved by the Committee for Ethics and Research of the São Paulo State University, School of Technology and Science—UNESP (CAAE: 54864716.8.0000.5402) and followed the Helsinki Declaration. The volunteers were informed about the procedures and objectives of this study and, after agreeing to participate, signed the consent form.

### Participants

Male patients of CRP who were previously diagnosed by their cardiologists with ischemic coronary artery disease and left ventricular ejection fraction higher than 50% were invited to participate in the study, after the previous analysis of the medical record regarding the inclusion/exclusion criteria.

Participants with less than 3 months of participation in CRP, those who were alcoholics and/or smokers, subjects with respiratory diseases, unstable angina, non-controlled hypertension, significant valvular disease, non-controlled metabolic disease, and/or neurological problems that could preclude the protocol execution were not included. The exclusion criteria were: abnormal hemodynamic responses during the cardiopulmonary exercise test^23^, presence of a series of RR intervals with less than 95% of sinus heartbeats^22^ and non-attendance to one of the protocol phases.

### Interventions

#### Cardiopulmonary exercise test

To define the exercise load and to evaluate the hemodynamic responses, the volunteers performed a maximum stress test on a treadmill (INBRASPORT), according to Bruce’s protocol, that was preceded by 5 min of warming-up at 2.5 km/h^24^. The test was conducted by a physician and was interrupted by voluntary exertion and/or ischemia signs and/or severe arrhythmia. To determine the oxygen uptake (VO_2_), the exhaled gases were analyzed by the Quark PFT (COSMED) system^25^ calibrated with volumes and gases of known concentration. The exercise load used in Phases II and III was set as 60–80% of the HR reached at the anaerobic threshold. For those who did not reach the anaerobic threshold during the test, the oxygen uptake peak was considered^23,26^. This intensity is considered as low-moderate, which is safe and commonly used in CRP^26^. Exercises performed above the anaerobic threshold may produce negative acute responses in subjects with CAD, such as metabolic acidosis, hyperventilation, and reduction in the capacity of performing the exercise^26^.

#### Control and hydration protocols

The protocols were performed between 1:00 and 6:00 pm to avoid the circadian variation and the temperature and humidity of the room were controlled (22–25 °C and 40–60%, respectively)^22^.

After volunteer arrival, a urine sample was collected to identify the hydration status. Also, body mass and height were measured with a digital scale (BALMAK) and a stadiometer (SANNY), respectively.

After these procedures, the volunteers remained in the orthostatic position for 10 min. Then, they performed 15 min of warming-up, composed by stretching and global exercises, followed by 40 min of treadmill exercise, that was stopped without a cool-down period. After this, volunteers remained on the treadmill in orthostatic position for 10 min performing the passive recovery. After recovery, another urine sample was collected, and the body mass was measured. The axillar temperature was measured with a thermometer (G-TECH) before and immediately after exercise.

During the HP, volunteers ingested four equal portions of mineral water (BONAFONT) every 10 min during the treadmill exercise, as done in other studies^18–20^.

#### Hydration condition

The volunteers were instructed to ingest 500 ml of water two hours before the protocols to ensure an initial hydrated state^27^. The amount of water administered during the HP was obtained through the body mass difference verified before and after the CP, considering that one gram is equivalent to one milliliter of fluid lost, as described by Armstrong et al.^28^ and applied by other studies^18–20^.

The dynamic hydration status before and after the protocols was assessed by the urine analysis (Combur 10 M ROCHE). The urine specific density was used as a marker of the hydration level, and values above 1.020 classified the volunteer as dehydrated^28^.

### Outcomes

#### Heart rate variability (HRV)

The vagal reactivation and the autonomic modulation were evaluated through HRV^29^, a non-invasive method based on the oscillations between consecutive RR intervals^30^ recorded through a HR monitor (POLAR ELECTRO)^31^.

To assess the vagal reactivation, the RMSSD index (root mean square of successive RR interval differences) was analyzed in 30-s intervals (RMSSD_30_) as proposed by Goldberger et al.^32^ and applied by other studies^14,15,33^. The last 30 s of the initial rest (M1), the last 30 s of the exercise (M2), and the first 2 min of the recovery, divided into four windows of 30 s each (M3, M4, M5, and M6), were considered.

This analysis was complemented by the evaluation of the autonomic modulation during the first 10 min of recovery. The last 5 min of the initial rest (REST) and the 10th-minute recovery divided into REC1 (0–5th minute) and REC2 (5th–10th minute) were considered. The HRV was analyzed in the time and frequency domains, as follows: LF (low-frequency component—0,04 to 0,15 Hz) and HF (high-frequency component—0,15 to 0,40 Hz) in milliseconds squared (ms^2^); LF/HF ratio; SDNN (standard deviation of all normal RR intervals); and RMSSD^30^.

The series of RR intervals were submitted to a digital filtration through the Polar Pro Trainer Software^®^ (version 5.0, Polar Inc., Kempele, Finland)^34^ and only the series with more than 95% of sinus heartbeat were included. Also, visual analysis was performed to ensure the absence of artifacts or cardiac arrhythmias that could interfere in the analysis^22^. The indexes were calculated with the software Kubios HRV—version 2.0^35^. This analysis was performed by an experienced and blinded researcher.

#### Heart rate recovery (HRR)

The parasympathetic reactivation after exercise was also indirectly evaluated by the HRR^29^. In order to determine the HRR, the HR was measured at three moments: at the peak of effort (HRpeak), at the first (HR1) and the second (HR2) minutes of recovery.

The HRpeak was defined as the mean of 5 RR intervals, considering two values before and two values after the HRpeak. The same procedure was used to obtain the HR1 and HR2. The HRR was defined by the difference between the HRpeak and HR1 (HRpeak—HR1 = HRR1) and between HRpeak and HR2 (HRRpeak—HR2 = HRR2)^36^. This procedure was also performed by an experienced and blinded researcher.

#### Rate of perceived exertion, recovery, and discomfort

The rate of perceived exertion (RPE) was evaluated by the Borg scale, an easy-to-use clinical tool commonly applied at the CRP to estimate the whole-body perceived effort, and it is known for indirectly correlating with the HR^37^. The Borg CR10 scale was used to define the perceived discomfort during the protocols. These scales were applied at the 10th minute of the rest, 15th and 35th minute of the exercise and 1st, 2nd, 3rd, 7th, and 10th minute of the recovery.

The rate of perceived recovery (RPR) was evaluated by a 10-point Likert scale, in which 1 corresponds to no recovery and 10 to fully recovered^38^ and it was applied during the recovery period.

### Statistical analysis

The normality of data was determined by the Shapiro–Wilk test. To body mass, urine specific density, and axillar temperature, the comparison between the moments of the same protocol were performed by two-tailed paired Student’s t-test or Wilcoxon test. For the analysis between the protocols, two-tailed unpaired Student’s t-test or Mann–Whitney test were performed.

The comparison of HRR, HRV, RPE, RPR, and Borg CR10 between the protocols and moments was performed by two-way repeated-measures ANOVA. The data were checked for sphericity violation using Mauchly’s test and the Greenhouse–Geisser correction was considered when the sphericity was violated.

To analyze the moments during the same protocol, the Bonferroni post hoc test for parametric distribution or Dunnet post hoc test for non-parametric distributions were applied. The partial eta-squared effect size (η^2^_P_) was calculated for the ANOVA results (small ≤ 0.05; medium between 0.06 and 0.13; large ≥ 0.14)^39^. The HRR1 and HRR2 were analyzed between groups by the two-tailed unpaired Student’s t-test or Mann–Whitney test and the intragroup analysis was performed by the two-tailed paired Student’s t-test or Wilcoxon test. The Cohen’s d effect size was calculated for these analyses (small < 0.50; medium between 0.50 and 0.70; large between 0.80 and 1.20; very large ≥ 1.30)^39^.

The statistical significance was set at 5%. The analyses were performed using IBM SPSS Statistics—version 22.0 based on a coded data sheet by a blinded researcher.

### Sample size

The sample size was based on the RMSSD index. The magnitude of significant difference assumed was 12 ms, considering a standard deviation of 16 ms^36^, with alfa risk of 5% and a beta risk of 80%, which resulted in a sample size of 28 volunteers. Considering the possible sample losses, we added 10% to the sample size calculated, totalizing 31 volunteers.

## Results

### Sample characterization and hydration condition

**Figure 1:**
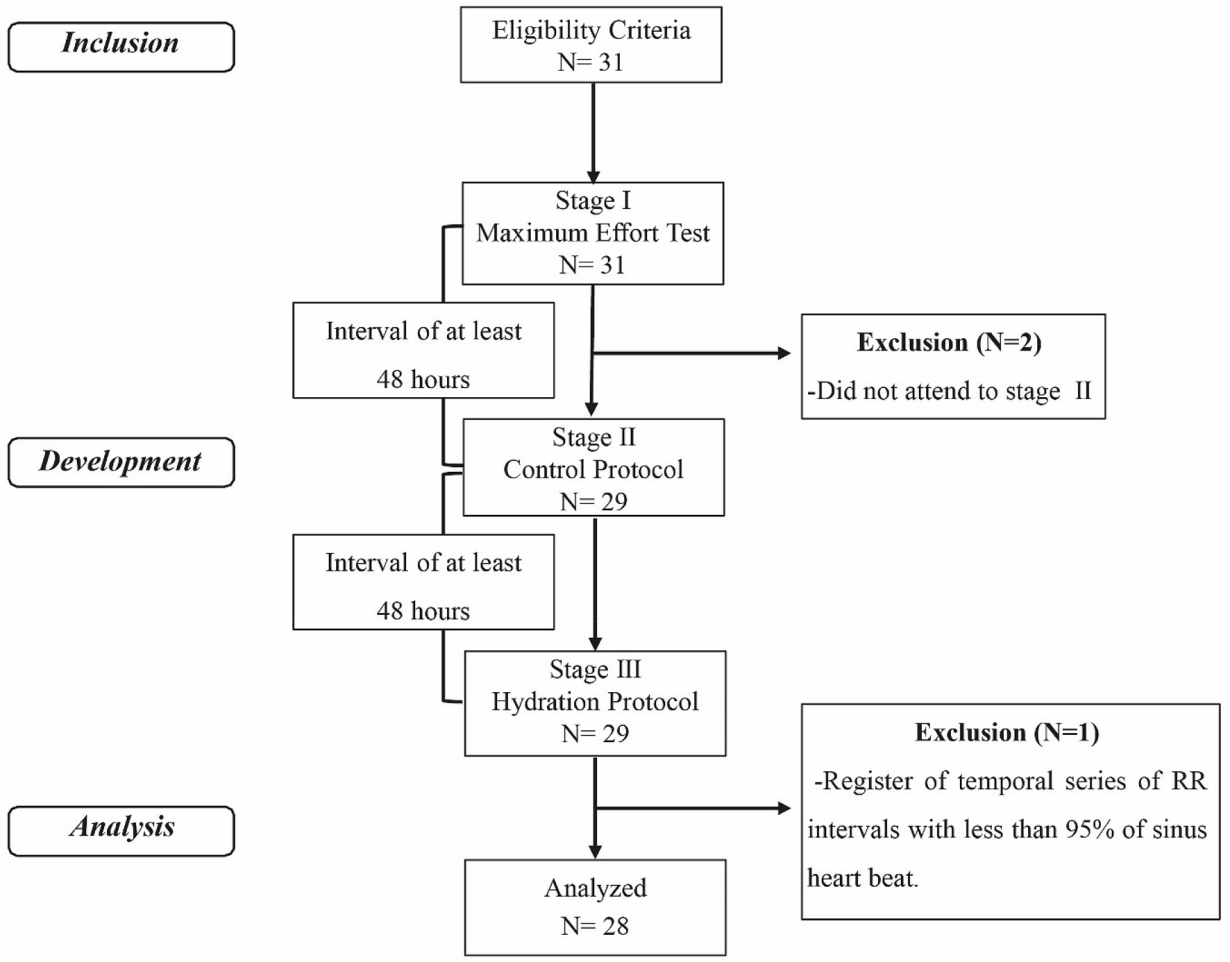
Protocol design.

In total, 31 subjects were recruited (Fig. 1). After sample losses, 28 subjects were analyzed (Table 1).

**Table 1:** Sample characterization (N = 28).

Variables	Mean ± SD	Minimum	Maximum
Age (years)	63.61 ± 8.48	45.00	83.00
Period of treatment in CR (years)	3.27 ± 3.33	0.27	11.98
Weight (kg)	80.42 ± 12.94	58.00	108.50
Height (m)	1.71 ± 0.05	1.58	1.81
BMI (kg/m^2^)	27.44 ± 4.25	20.62	39.37
SBP (mmHg)	120.36 ± 8.38	110.00	150.00
DBP (mmHg)	77.50 ± 8.44	60.00	100.00
Resting HR (bpm)	72.50 ± 11.64	55.00	96.00
Work load (km/h)	4.92 ± 0.69	3.30	6.00
**Maximal stress test**			
VO_2_peak (ml/min/kg)	26.10 ± 5.27	14.28	37.27
Maximum HR (bpm)	141.57 ± 20.70	103.00	173.00

**Risk factors N(%)**			
Diabetes	11 (39.3%)		
Dyslipidemia	18 (64.3%)		
Ex-smoker	15 (53.6%)		
High blood pressure	25 (89.3%)		
Family history	20 (71.4%)		
Obesity	5 (17.9%)		
**Medicines N(%)**			
Anti-platelet drugs	22 (78.6%)		
ARA II	10 (35.7%)		
Ca^+^ channel blockers	8 (28.6%)		
K^+^ channel blockers	1 (3.6%)		
Beta bockers	18 (64.3%)		
Diuretics	7 (25.0%)		
Statins	23 (82.1%)		
Hypoglycemic agents	7 (25.0%)		
ACE inhibitor	5 (17.9%)		
Others	8 (28.6%)		
Vasodilator	2 (7.1%)		

*CR* cardiac rehabilitation, *kg* kilogram, *m* meters, *kg/m*^*2*^ kilogram/meter^2^, *mmHg* millimeters of mercury, *bpm* beats per minute, *ml/min/kg* milliliter/minute/kilogram, *km/h* kilometer/hour, *BMI* body mass index, *SBP* systolic blood pressure, *DBP* diastolic blood pressure, *HR* heart rate, *VO*_*2*_*peak* peak oxygen consumption, *ARA II* angiotensin II receptor antagonists, *ACE* angiotensin-converting enzyme, *Ca*^+^ calcium, *K*^+^ potassium.

The body mass, axillar temperature, and urine specific density values are presented in Table 2. A significant reduction of the body mass was observed at the end of CP (0.345 ± 0.121 kg). Significant differences between the protocols for axillary temperature and specific density were not found.

**Table 2:** Sample characterization in relation to body mass, axillar temperature, and urine specific density before and after protocols (N = 28).

Variables	Moment	Control protocol	Hydration protocol
Body mass (kg)	Initial	80.98 ± 12.71	80.98 ± 12.82
	Final	80.64 ± 12.70^a^	80.97 ± 12.80
Axillar temperature (°C)	Initial	35.20 ± 0.54	35.02 ± 0.74
	Final	34.90 ± 0.97	34.82 ± 0.98
Urine specific density	Initial	1.016 ± 0.004	1.020 ± 0.035
	Final	1.016 ± 0.004	1.014 ± 0.003

Mean ± standard deviation; *kg* kilogram, °C degree Celsius.

^a^Difference between the initial body mass in the control protocol; *p*_value_ = 0.000 (two-talied paired student t test).

### Heart rate variability and heart rate recovery analysis

**Figure 2:**
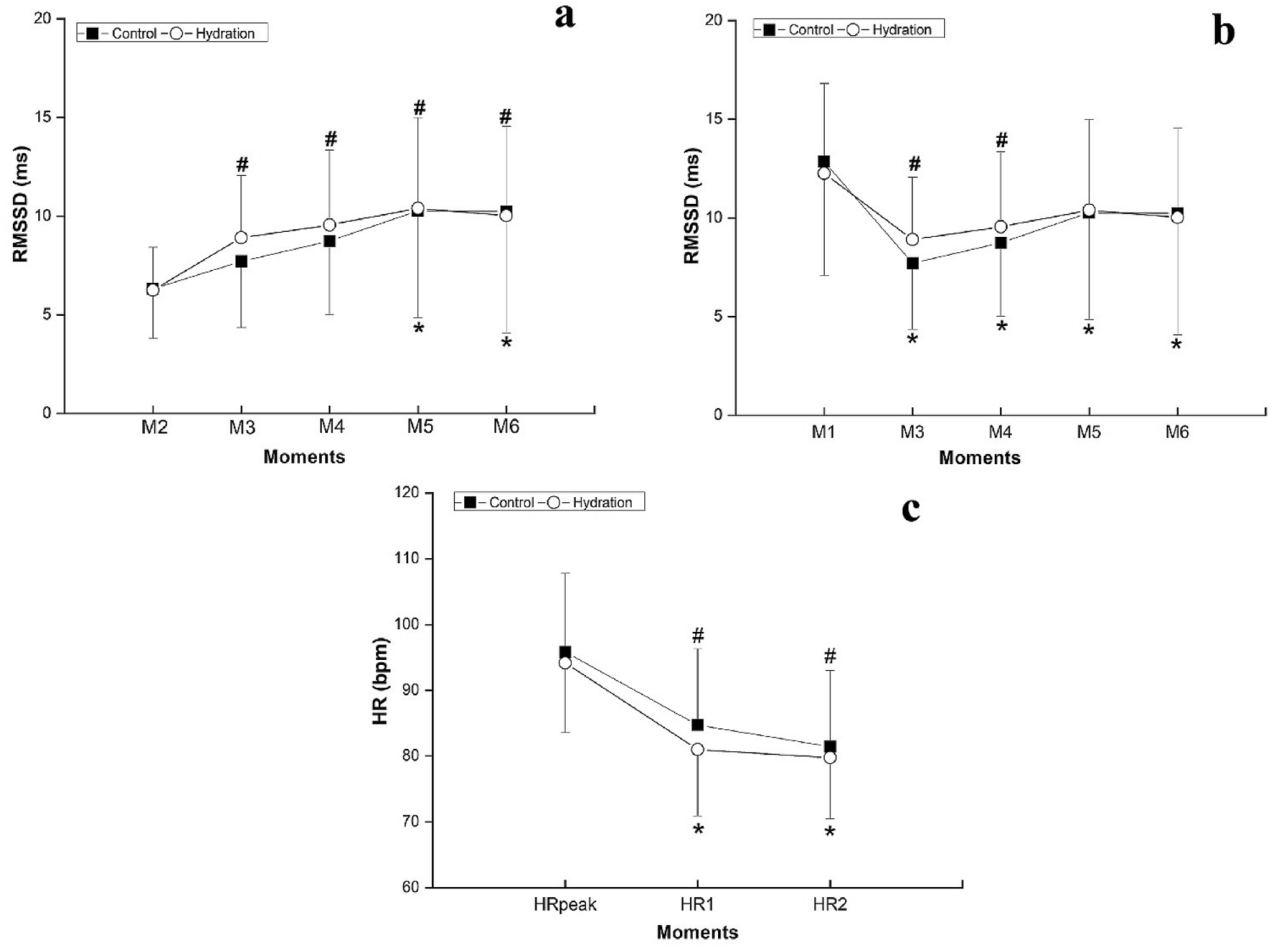
Comparison of RMSSD_30_ at exercise (**a**) and initial rest (**b**) and comparison between HRpeak and HR1 and HR2 (**c**). Legend: M1 = last 30 s of initial rest; M2 = last 30 s of exercise; M3 = 0–30 s, M4 = 30–60 s, M5 = 60–90 s, M6 = 90–120 s of recovery; *HRpeak* heart rate at the peak of exercise, *HR1* heart rate after 1 min of recovery, *HR2* heart rate after 2 min of recovery. *Difference between exercise (**a**)/initial rest (**b**) and recovery on control protocol (*p*_value_ < 0.001); #Difference between exercise (**a**)/initial rest (**b**) and recovery on hydration protocol (*p*_value_ < 0.05).

The RMSSD_30_ during the early recovery period was compared with the values at rest (Fig. 2b) and at the end of the exercise (Fig. 2a), to address the parasympathetic return to the rest values and the vagal reactivation, respectively. For both analysis, there were no significant differences between the protocols (2a: *p*_value_ = 0.671; η^2^_P_ = 0.003—small effect size/2b: *p*_value_ = 0.791; η^2^_P_ = 0.001—small effect size) and at the moments vs protocols interaction (2a: *p*_value_ = 0.610; η^2^_P_ = 0.012—small effect size/2b: *p*_value_ = 0.469; η^2^_P_ = 0.016—small effect size). However, there were differences between the moments (2a: *p*_value_ < 0.001; η^2^_P_ = 0.267—large effect size/2b: *p*_value_ = 0.001; η^2^_P_ = 0.237—large effect size).

For the HR analysis (Fig. 2c), no significant differences were found between the protocols (*p*_value_ = 0.583; η^2^_P_ = 0.013—small effect size) and at the moments vs protocols interaction (*p*_value_ = 0.305; η^2^_P_ = 0.021—small effect size). However, between the moments significant differences were found (*p*_value_ = 0.001; η^2^_P_ = 0.781—large effect size).

In the CP significant differences between HRR1 (11.11 ± 6.57) and HRR2 (14.40 ± 5.41; *p*_value_ < 0.0001; Cohen’s d = 0.55—medium effect size) were observed. However, for HP there were no differences between HRR1 (13.18 ± 8.02) and HRR2 (14.41 ± 6.60; *p*_value_ = 0.132; Cohen’s d = 0.17—small effect size). Comparisons of HRR1 and HRR2 between the protocols showed no significant statistical differences (HRR1: *p*_value_ = 0.237; Cohen’s d = 0.28—small effect size/HRR2: *p*_value_ = 0.554; Cohen’s d = 0.00—small effect size).

The results of the 5 min analysis of SDNN, RMSSD, LF, HF, LF/HF are presented in Table 3. There were no statistical differences between the protocols.

**Table 3:** Heart rate variability behavior for the 5-min windows in both protocols (N = 28).

Variable	Protocol	REST	REC1	REC2	ANOVA results *p*_value_ (η^2^_P_)
SDNN	Control	29.31 ± 10.88	38.88 ± 13.34***	29.10 ± 10.46	M: 0.000 (0.335)
	Hydration	31.43 ± 11.94	40.18 ± 12.26***	31.90 ± 11.94	I: 0.864 (0.003)
RMSSD	Control	14.07 ± 6.68	9.96 ± 4.44***	10.73 ± 4.50***	M: 0.000^a^ (0.370)
	Hydration	14.91 ± 6.35	10.94 ± 4.09***	11.90 ± 5.20***	I: 0.953 (0.001)
LF (ms^2^)	Control	232.82 ± 194.8	219.32 ± 310.41	321.14 ± 266.42	M: 0.013 (0.079)
	Hydration	244.78 ± 194.33	299.53 ± 274.67	361.32 ± 341.09	I: 0.614 (0.009)
HF (ms^2^)	Control	66.25 ± 72.20	37.64 ± 50.78*	32.93 ± 28.20	M: 0.000 (0.235)
	Hydration	73.14 ± 64.95	36.18 ± 29.17***	43.68 ± 48.17	I: 0.627 (0.009)
LF/HF	Control	6.22 ± 4.68	7.46 ± 5.05	14.22 ± 17.72***	M: 0.000 (0.204)
	Hydration	5.45 ± 4.57	9.90 ± 7.99**	11.32 ± 8.54***	I: 0.133 (0.037)

Mean ± standard deviation. Bold values: significantly different from REST.

*ms*^*2*^ millisecond squared, *REST* last 5 min of initial rest, *REC1* first 5 min of recovery, *REC2* 5th–10th minute of recovery, *M* moments, *I* interaction moments versus protocol.

***p_value_ < 0.001; **p_value_ < 0.01; *p_value_ < 0.05.

### Rate of perceived exertion and recovery analysis

**Figure 3:**
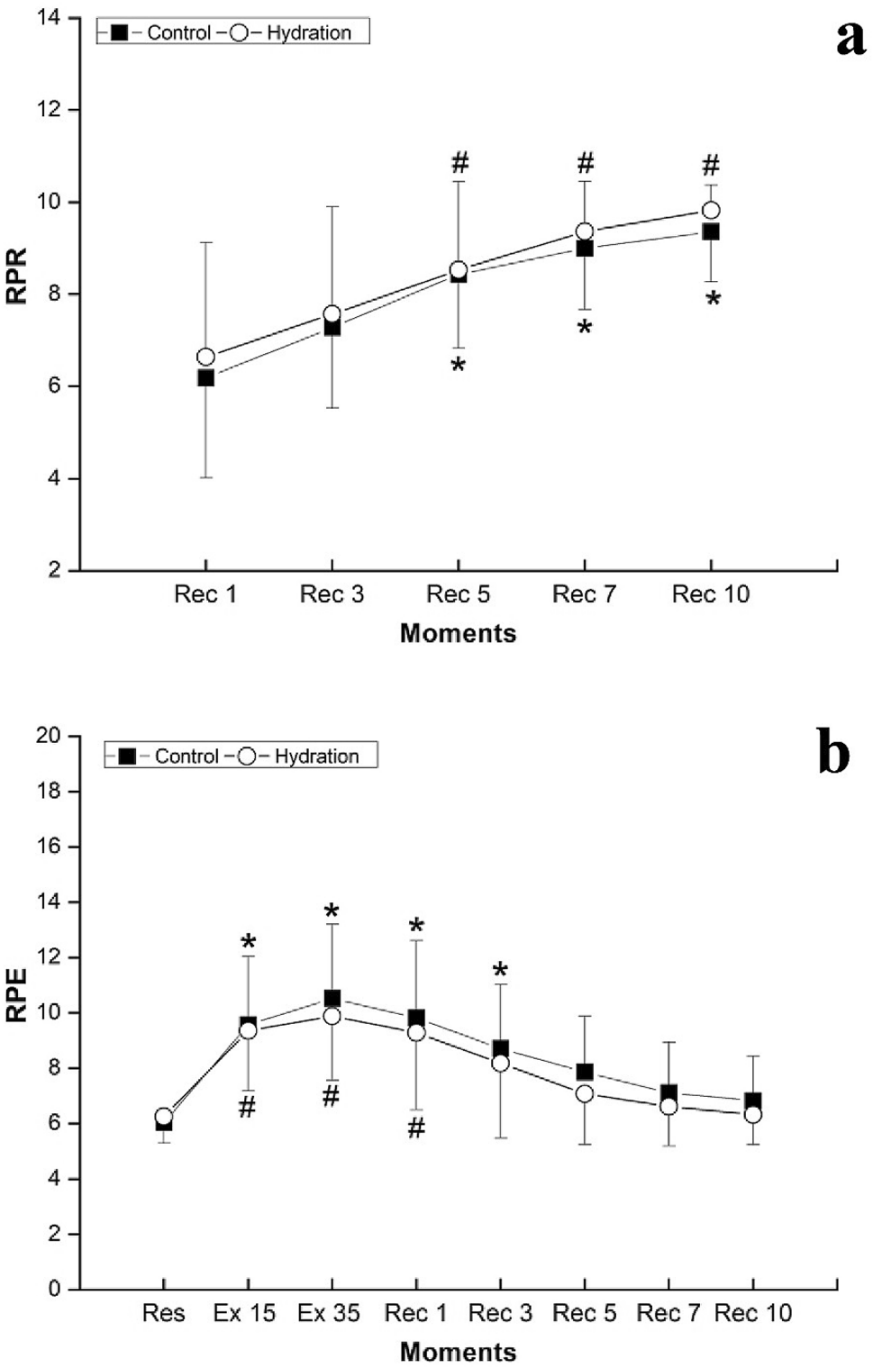
Mean and standard deviation for the rate of perceived recovery (**a**) and exertion (**b**) scale in both protocols. Legend: Rec 1 = 1st minute of recovery; Rec 3 = 3rd minute of recovery; Rec 5 = 5th minute of recovery; Rec 7 = 7th minute of recovery; Rec 10 = 10th minute of recovery; Res = initial rest; Ex 15 = 15th of treadmill exercise; Ex 35 = 35th minute of treadmill exercise; *RPR* rate of perceived recovery; *RPE* rate of perceived exertion. *Difference between Rec 1 and other moments of recovery (**a**)/initial rest and recovery (**b**) of control protocol (*p*_value_ < 0.001); #Difference between Rec 1 and other moments of recovery (**a**)/initial rest and recovery (**b**) of hydration protocol (*p*_value_ < 0.001).

For the RPE and RPR (Fig. 3), there were no significant differences between the protocols (RPE: *p*_value_ = 0.302, η^2^_P_ = 0,020—small effect size; RPR: *p*_value_ = 0.390, η^2^_P_ = 0.014—small effect size) and for the moments vs protocols interaction (RPE: *p*_value_ = 0.360, η^2^_P_ = 0.018—small effect size; RPR: *p*_value_ = 0.743, η^2^_P_ = 0.005—small effect size). However, for the moments, differences were observed for both scales (RPE: *p*_value_ = 0.001, η^2^_P_ = 0.519—large effect size; RPR: *p*_value_ = 0.001, η^2^_P_ = 0.607—large effect size).

## Discussion

The results revealed that the hydration did not promote significant differences between the protocols for the vagal reactivation, autonomic modulation recovery, RPE, and RPR after a moderate-intensity session of exercise. However, small anticipation of vagal reactivation, autonomic modulation recovery, and RPE was observed when the hydration protocol was performed.

It is reported that hypohydration/dehydrated states promote a linear increase in body temperature^40^. However, it was not observed in the present study. This suggests that the exercise model used in CRP, even without fluid replacement, does not promote significant hypohydration/dehydration, which contributes to the safety of these programs, considering the deleterious effects of dehydration in the cardiovascular system. However, the hydration strategy used in our study was efficient to avoid fluid losses, as the body mass did not change in the HP.

The analysis of the RMSSD_30_, a marker of the parasympathetic modulation^30^, demonstrated the physiological gradual increase of vagal activity during recovery^8^. In the HP, the vagal reactivation may have occurred sooner, since, after the first minute of recovery, the RMSSD_30_ was no longer significantly different from the rest and, at the first 30 s of recovery the parasympathetic modulation was significantly higher than at the exercise. In the CP, the RMSSD_30_ did not return to the rest values and only became significantly higher than the exercise after the first minute of recovery. However, these results should be analyzed with caution due to the non-significant difference between the protocols and small effect sizes.

After exercise, the decrease of HR depends on the vagal reactivation^8^. The higher HRR1 observed at HP, even without significant difference between protocols, suggests a small acceleration of the parasympathetic reactivation at HP, in accordance with the RMSSD_30_ results_._ The low HRR1 and high HRR2 in the CP evidence a slower HR recovery when the volunteers did not ingest water. This response was not observed in HP, where HRR1 was as high as HRR2. Also, this faster HR reduction could explain the faster reduction of the RPE after exercise with water intake, since it is reported that the RPE scale correlates to the HR^37^.

The HRV indexes analyzed in 5-min windows had a physiological and similar response in both protocols. The significant increase of SDNN, which represents the global modulation at REC1 and its return to the rest value at REC2, in both protocols may be related to the vagal reactivation, evidenced in this study through the RMSSD_30_. In addition, the passive recovery in the orthostatic position for 10 min was insufficient to promote the total recovery of the parasympathetic modulation, even with the hydration.

This is the first study to investigate the influence of hydration in the immediate autonomic recovery of CAD subjects. This topic was previously studied in health and young males, who ingested 500 ml of water in a single dose immediately after moderate and high-intensity exercises^14,15,17^ and had a positive effect on the vagal reactivation and autonomic modulation recovery.

However, in our study, the volunteers ingested an individualized amount of water in fractionated doses throughout the exercise, as suggested by the American College of Sports Medicine^23^. The total amount of water ingested was 0.345 ± 0.11L, which was sufficient to avoid the fluid losses and, even if not significant, it promoted small anticipation of the recovery. However, if it is enough to minimize the appearance of sudden events during the recovery period is still an aspect that needs to be further studied.

Furthermore, another factor that may be responsible for the low effect of the hydration strategy used in our study is the exercise intensity proposed. The exercise in our experiment was performed bellow the anaerobic threshold, that classifies it as low-moderate intensity^26^, and it is known in the literature that, in healthy and young subjects, the higher the exercise intensity, the higher the sweating rate, fluid losses^40^, and the cardiovascular^41^ and autonomic^12^ perturbations. Thus, this aspect may justify the low effect of the hydration in the vagal reactivation, HRR and RPE.

As a study limitation, the randomization of the protocols was not possible because the amount of water ingested was obtained by assessing body mass loss after the CP. However, all participants were familiar with the proposed exercise protocol, so we believe that the exercise model performed in the study did not influence the outcomes analyzed.

Despite the small and non-significant effects between protocols found in the present study in the immediate recovery period of a cardiac rehabilitation session, the investigation of new hydration strategies capable of accelerating the recovery in CAD subjects is important and should be encouraged, since those individuals present an autonomic impairment^1^ and have a less efficient recovery after exercise. Therefore, from our preliminary results, new studies that include larger samples and that insert randomization of different hydration strategies during exercise, can be designed. In addition, it may be interesting to analyze a longer recovery period to understand the impact of hydration strategies on the autonomic behavior in the recovery period of CAD patients.

## Conclusions

The hydration protocol performed did not promote significative changes in the vagal reactivation, autonomic modulation recovery, and rate of perceived exertion and recovery, when compared to the control protocol. However, the hydration protocol avoided the fluid losses induced by exercise and promoted small anticipation of vagal reactivation and autonomic modulation recovery and a small reduction at the rate of perceived exertion.
